# Common Themes and Uncertainties in Management of Secondary Polycythaemia: An International Clinician Survey of Practice

**DOI:** 10.1002/jha2.70171

**Published:** 2025-10-27

**Authors:** Phillip LR Nicolson, Midhat Asif, Richard J Buka, Peter Dyer, Eman Hassan, Claire N Harrison, Bernard D Maybury, Andrew J Doyle

**Affiliations:** ^1^ Department of Cardiovascular Sciences College of Medicine and Health University of Birmingham Birmingham UK; ^2^ Department of Clinical Haematology Queen Elizabeth Hospital Birmingham UK; ^3^ HaemSTAR UK; ^4^ Department of Clinical Haematology Royal Stoke Hospital Stoke‐on‐Trent UK; ^5^ Department of Clinical Haematology Guy's and St Thomas’ NHS Foundation Trust London UK

## Abstract

**Introduction:**

Secondary and idiopathic polycythaemia is far more common than polycythaemia vera. Whilst venesection for polycythaemia vera has a robust evidence base, the data supporting this treatment for secondary and/or idiopathic polycythaemia are limited to small single‐arm studies showing transient improvement in symptoms or physiologic endpoints. As a result the British Society for Haematology Guideline for the management of specific situations in polycythaemia vera and secondary erythrocytosis suggests *considering* venesection in patients with hypoxic lung disease with Hct > 0.56 or to a target Hct < 0.55 in those with idiopathic polycythaemia. We hypothesised that this paucity of evidence results in widespread variation in management of secondary polycythaemia.

**Methods:**

To assess attitudes and practice in the treatment of secondary and idiopathic polycythaemia we designed an international clinician survey to evaluate current practice and define the point of clinical equipoise at which clinicians would be content to enter patients into a randomised venesection study. This survey was distributed using the wide‐reaching HaemSTAR research network.

**Results:**

A total of 123 clinicians responded, of which 90 were experienced senior clinicians. 62% reported not routinely offering regular venesection whereas 38% of respondents did. Those considering venesection rose to ∼2/3 of respondents in specific circumstances such as polycythaemia‐related symptoms, previous arterial or unprovoked venous thrombosis. Respondents were more likely to offer venesection to patients with idiopathic‐ compared to androgen‐ or hypoxia‐driven polycythaemia. Of those who would venesect, most would use a threshold Hct ≥ 0.55, with a target Hct < 0.55 but there was significant variability.

**Conclusions:**

Our survey showed considerable variability in venesection practice for patients with secondary or idiopathic polycythaemia, probably reflecting the paucity of the evidence base. There was widespread support for a trial of venesection vs observation in secondary polycythaemia and this survey has helped to define the threshold Hct at which clinical equipoise exists.

1

Polycythaemia is characterised by an increase in the haematocrit (Hct) above the sex specific reference range. There is a robust evidence base for the management of clonal polycythaemia (polycythaemia vera, [PV]) that includes a large clinical trial showing venesection to a target Hct of < 0.45 results in reduced thrombosis and cardiovascular death [1]. This has resulted in clear management guidelines for PV [2]. Only 2% of polycythaemia is clonal; however, with the remaining 98% either secondary to other medical conditions such as hypoxic lung disease or testosterone treatment or idiopathic [3]. Notably secondary polycythaemia during androgen replacement is an increasingly encountered clinical scenario as hormonal therapy for gender transition and non‐therapeutic testosterone use becomes more common. The evidence for benefit of venesection in secondary polycythaemia is limited to small single‐arm studies showing transient improvement in symptoms or physiologic endpoints but it could be argued that these studies lack meaningful clinical endpoints [4, 5, 6, 7]. Conversely, it is well documented that patients with chronic obstructive pulmonary disease (COPD) and anaemia have worse survival than those with COPD and polycythaemia [8]. In the absence of a robust evidence base, only very limited (Grade 2C) guidance is available for treatment of secondary polycythaemia [9]. These guidelines suggest *considering* venesection in patients with hypoxic lung disease with Hct > 0.56 or to a target Hct < 0.55 in those with idiopathic polycythaemia [9]. We hypothesised that this paucity of evidence results in widespread variation in management of secondary polycythaemia. As a result we designed an international survey to evaluate current practice and define the point of clinical equipoise at which clinicians would be content to enter patients into a randomised venesection study. This survey included questions about respondent practice and their management of hypothetical clinical scenarios. The survey (see Supporting Information) was shared via the wide‐reaching HaemSTAR network [10].  The clinical scenarios described four male patients with secondary or idiopathic polycythaemia (secondary causes were COPD, obstructive sleep apnoea [OSA] and androgen supplementation) where the diagnosis of polycythaemia was robust, who had been medically optimised and were otherwise identical. Respondents were asked to state whether they would venesect, and what Hct thresholds and targets they would use. They were also asked whether any aspects in the medical history or particular symptoms would influence this decision and whether they would consider enrolling their patients with secondary or idiopathic polycythaemia into a prospective randomised clinical trial of target‐guided venesection versus no treatment.

Table S1 shows respondents’ demographics including grade and location and whether they regularly venesect or indeed give cytoreductive pharmacological therapy to people with secondary polycythaemia. A total of 123 clinicians responded to the survey, of which 101 were from the UK. Among them, 90 respondents (73%) were senior clinicians (consultants). Results from the consultant and overall cohorts were similar. Thirty eight percent of total respondents and consultants regularly offer venesection, whereas 62% do not. Overall 10% of respondents and 8% of consultants also offer cytoreduction for management of secondary or idiopathic polycythaemia.

Proposed management of the four hypothetical cases are shown in Figure 1 and Table S2 (for consultants) and Figure S1 and Table S3 (for all respondents). Broadly respondents were split with approximately 2/3 opting to venesect in specific circumstances, 1/3 never venesecting and only a minority routinely venesecting. Respondents were more likely to routinely venesect patients with idiopathic polycythaemia when compared to androgen‐ or hypoxia‐driven polycythaemia (Figure 1A, Figure S1A, Tables S2 and S3). Of those consultants who would venesect the four hypothetical patients, most (50%–67%) suggested a venesection threshold of Hct ≥ 0.55 (probably reflecting extrapolation from BSH Guidelines) [9] but a significant minority (15%–35%) suggested Hct ≥ 0.6 (Figure 1Bi). The majority of consultants (40%–56%) would venesect to a target Hct < 0.55, but a significant minority (23%–28%) would use a target Hct < 0.52 (Figure 1Bii) (probably reflecting the BSH criterion for PV diagnosis) [2]. Eighty eight percent of consultants would start venesection or lower their venesection thresholds/targets based on clinical features/symptoms (Figure 1Ci). There was broad agreement (63%–85% of consultants who would consider venesection) that a history of arterial thrombosis, unprovoked venous thrombosis or symptoms related to polycythaemia would push respondents to venesect or lower their venesection thresholds/targets (Figure 1Ci, Figure S1Ci). The most common polycythaemia related symptoms cited were headache and visual disturbance (Figure 1Cii, Figure S1Cii). In case practice variability resulted from inclusion of respondents from disparate healthcare systems, we repeated these analyses including only UK respondents. No differences in responses were found when compared to the overall cohort (Table S3, Figure S2). Eighty five percent of respondents indicated they would be willing to consider entering patients with secondary polycythaemia into a randomised controlled trial of venesection with 75% of these agreeing a venesection threshold of Hct > 0.6 unless there was a history of arterial or venous thrombosis in which case > 0.55 would be used (Table S5).

**FIGURE 1:**
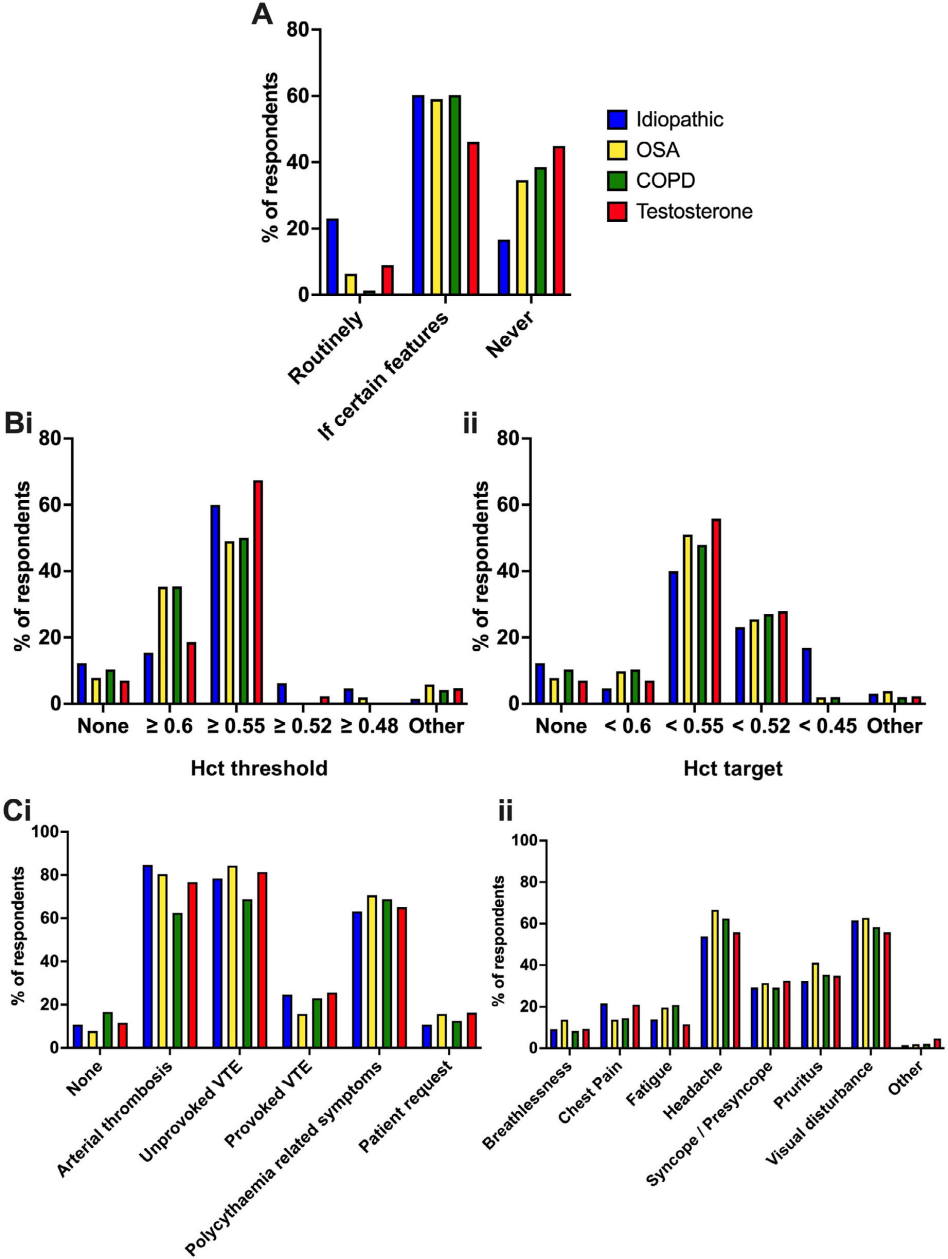
Management suggestions on four hypothetical cases of secondary polycythaemia by respondents self identifying as senior/experienced physicians. In all cases the assumptions made were that the diagnoses were secure and the management of the underlying cause had been medically optimised. (A) Percentage of total senior respondents who would routinely venesect, venesect only in certain circumstances or never venesect each of the cases. (Bi) Percentage of total senior respondents who would use different venesection thresholds. ii) Percentage of total senior respondents who would use different venesection targets. (C) Percentage of senior respondents indicating that clinical features would alter their management who would initiate venesection or alter threshold/target i) in the presence of certain features in the past medical history, ii) in the presence of certain symptoms.

These data show that there is considerable variation in practice in management of patients with secondary polycythaemia. This is probably due to the paucity of evidence on which to base management decisions. There is more of a willingness to venesect those with idiopathic polycythaemia. This may reflect uncertainty as to whether patients with idiopathic polycythaemia could have an occult, clonally‐driven polycythaemia. These data also show that there is an uncertainty about whether to instigate venesection at Hct ≥ 0.6 or ≥ 0.55, or not at all, and whether the target Hct should be < 0.55 or < 0.52. There is broad agreement; however, that a history of arterial thrombosis or unprovoked venous thrombosis, or ongoing symptoms of headache and visual disturbance would lower Hct thresholds and targets.

These data, along with that of our accompanying manuscript [11], demonstrate both the need to collect more robust clinical data in patients with secondary and idiopathic polycythaemia, and a willingness to participate in this evidence generation. These data help define the point of clinical equipoise and give important information to help design and power a prospective randomised controlled trial in this area.

## Author Contributions

P.L.R.N., R.J.B., C.N.H., B.D.M. and A.J.D. conceived the study. P.L.R.N., R.J.B., P.D., C.N.H., B.D.M. and A.J.D. designed the data collection tool. P.L.R.N., M.A. and E.H. analysed data. P.L.R.N. and M.A. wrote the manuscript. P.L.R.N., M.A., R.J.B., P.D., E.H., C.N.H., B.D.M. and A.J.D. edited the manuscript.

## Funding

The authors have nothing to report.

## Conflicts of Interest

The authors declare no conflicts of interest.

## Ethics Statement

The authors have nothing to report.

## Consent

The authors have nothing to report.

## Supporting information




**Supporting File 1**: jha270171‐sup‐0001‐SuppMat.pdf

**Supporting File 2**: jha270171‐sup‐0002‐SuppMat.docx

## Data Availability

All data can be made available upon reasonable request to the corresponding author.
