# Supplementary material for: Common Themes and Uncertainties in Management of Secondary Polycythaemia: An International Clinician Survey of Practice
Source: EJHaem. 2025 Oct 27;6(6):e70171. doi: 10.1002/jha2.70171 (PMC12558438; doi:10.1002/jha2.70171)
Supplement: Supplementary file 1 — Supporting File 1: jha270171‐sup‐0001‐SuppMat.pdf [file JHA2-6-e70171-s002.pdf]

Departmental Venesection Practice

THANK YOU VERY MUCH FOR GIVING UP YOUR TIME TO FILL OUT THIS SURVEY

The British Society of Haematology Guidelines on the management of secondary polycythaemia (McMullin et al. BJHaem 2018 (https://doi.org/10.1111/bjh.15647) suggest that:

- For idiopathic polycythaemia: haematocrit (Hct) can be controlled with venesection in selected cases with target Hct based on thrombotic history and risk factors (GRADE 2C)
- For polycythaemia related to hypoxic pulmonary disease: patients who are symptomatic as a result of hyperviscosity or have a Hct > 0.56 should be considered for venesection to reduce this to 0.50-0.52 (GRADE 2C)

The low grading of the recommendations reflects the paucity of evidence on which they are based. We believe there is an opportunity to establish a better evidence base through the running of a randomised clinical trial on venesection in these patients.

This survey is designed to:

- Ascertain whether there is a geographical variability in practice for offering venesection to patients with secondary or idiopathic polycythaemia.
- Establish if there is a point of clinical equipoise where treating clinicians are unsure whether venesection is of benefit or not in patients with secondary or idiopathic polycythaemia.
- Gauge whether there is sufficient interest in this area to start setting up a randomised controlled trial.

This first set of questions aims to answer point (1) above.

When you have finished, please click 'Submit' and you will be taken to the next part of the survey which uses three clinical vignettes to try and judge equipoise.

Where do you work?	◯ UK & Ireland<br>◯ Non-UK/Ireland
In which hospital / clinic do you work?	______________________
At what level do you work?	◯ Consultant / Attending<br>◯ Clinical Nurse Specialist<br>◯ Specialty Doctor<br>◯ Registrar / Fellow<br>◯ Senior House Officer / Senior Resident<br>◯ Foundation Doctor / Junior Resident<br>◯ Medical Student<br>◯ Other
if other, please specify	______________________
Do you routinely venesect patients for idiopathic and secondary polycythaemia?	◯ Yes<br>◯ No
Do your colleagues routinely venesection patients for idiopathic and secondary polycythaemia?	◯ Yes<br>◯ No
Approximately how many venesections per month take place in your department for idiopathic and secondary polycythaemia?	______________________
In your department, do patients receive cytoreduction (eg with hydroxycarbamide) for idiopathic and secondary polycythaemia?	◯ Yes<br>◯ No

We acknowledge that the following is an unusual question. Why does your department venesect but not cytoreduce patients with idiopathic and secondary polycythaemia? __________________________________

Case Vignettes

Please find below four clinical vignettes.

These have been designed to establish if there is a point of clinical equipoise where treating clinicians are unsure whether venesection is of benefit or not in patients with secondary or idiopathic polycythaemia.

Once you have finished please click 'Submit' at the bottom and you will be taken to the final, very short (!), part of the survey about willingness to participate in a randomised trial

---

In the following scenarios all patients:

- Are male
- Have raised haematocrit and persistent, true erythrocytosis
- Are negative for JAK2 V617F and JAK2 exon 12 mutations
- Have a non-suppressed serum erythropoietin
- Do not have splenomegaly
- Do not have polycythaemia related symptoms
- Do not have any other medical history

Please indicate the management you would recommend in each case.

---

1. For a patient with polycythaemia secondary to obstructive sleep apnoea following optimisation with continuous positive airway pressure (CPAP):

Would you offer venesection?

◯ Yes, I would routinely offer venesection.
◯ Yes, I would offer venesection for some patients depending on certain clinical features.
◯ No I would not offer venesection in any circumstances.

What is your routine threshold haematocrit for starting venesection?

◯ No threshold, I would not routinely offer venesection
◯ Hct ≥ 0.6
◯ Hct ≥ 0.55
◯ Hct ≥ 0.52
◯ Hct ≥ 0.48
◯ Other (please specify)

Please specify threshold haematocrit at at or above which you would offer venesection.

____

What is your target haematocrit after venesection?

◯ Target Hct < 0.6
◯ Target Hct < 0.55
◯ Target Hct < 0.52
◯ Target Hct < 0.45
◯ Other (please specify)

Please specify the target haematocrit, below which you would be satisfied that venesection was adequate.

____

Would any of the following features lower your haematocrit threshold / target or push you into considering venesection for this patient?

History of arterial thrombosis / venous thrombosis / polycythaemia related symptoms / patient request

◯ Yes
◯ No

If so, which? (please tick all that apply)	☐ History of arterial thrombosis<br>☐ History of unprovoked venous thrombosis<br>☐ History of provoked venous thrombosis<br>☐ Polycythaemia related symptoms<br>☐ Patient request
Which polycythaemia related symptoms would influence your decision to offer venesection?	☐ Abdominal pain<br>☐ Breathlessness<br>☐ Chest pain<br>☐ Fatigue<br>☐ Headache<br>☐ High blood pressure<br>☐ Light-headedness or dizziness<br>☐ Pruritis<br>☐ Visual disturbance<br>☐ Other (please specify)
Please specify 'other' polycythaemia related symptoms	__________________________

2. For a patient with polycythaemia secondary to COPD following optimisation after respiratory consultation:

Would you offer venesection?	◯ Yes, I would routinely offer venesection.<br>◯ Yes, I would offer venesection for some patients depending on certain clinical features.<br>◯ No I would not offer venesection in any circumstances.
What is your routine threshold haematocrit for offering venesection?	◯ No threshold, I would not routinely offer venesection<br>◯ Hct ≥ 0.6<br>◯ Hct ≥ 0.55<br>◯ Hct ≥ 0.52<br>◯ Hct ≥ 0.48<br>◯ Other (please specify)
Please specify threshold haematocrit at or above which you would offer venesection.	__________________________
What is your target haematocrit after venesection?	◯ Target Hct < 0.6<br>◯ Target Hct < 0.55<br>◯ Target Hct < 0.52<br>◯ Target Hct < 0.45<br>◯ Other (please specify)
Please specify the target haematocrit, below which you would be satisfied that venesection was adequate.	__________________________
Would any of the following features lower your haematocrit threshold / target or push you into considering venesection for this patient?<br><br>History of arterial thrombosis / venous thrombosis / polycythaemia related symptoms / patient request	◯ Yes<br>◯ No

If so, which? (please tick all that apply)	☐ History of arterial thrombosis<br>☐ History of unprovoked venous thrombosis<br>☐ History of provoked venous thrombosis<br>☐ Polycythaemia related symptoms<br>☐ Patient request
Which polycythaemia related symptoms would influence your decision to offer venesection?	☐ Abdominal pain<br>☐ Breathlessness<br>☐ Chest pain<br>☐ Fatigue<br>☐ Headache<br>☐ High blood pressure<br>☐ Light-headedness or dizziness<br>☐ Pruritis<br>☐ Visual disturbance<br>☐ Other (please specify)
Please specify 'other' polycythaemia related symptoms	__________________________________

3. For a patient with polycythaemia secondary to testosterone supplementation where the dose has been optimised for symptoms:

Would you offer venesection?	◯ Yes, I would routinely offer venesection.<br>◯ Yes, I would offer venesection for some patients depending on certain clinical features.<br>◯ No I would not offer venesection in any circumstances.
What is your routine threshold haematocrit for starting venesection?	◯ No threshold, I would not routinely offer venesection<br>◯ Hct ≥ 0.6<br>◯ Hct ≥ 0.55<br>◯ Hct ≥ 0.52<br>◯ Hct ≥ 0.48<br>◯ Other (please specify)
Please specify threshold haematocrit at or above which you would offer venesection.	__________________________________
What is your target haematocrit after venesection?	◯ Target Hct < 0.6<br>◯ Target Hct < 0.55<br>◯ Target Hct < 0.52<br>◯ Target Hct < 0.45<br>◯ Other (please specify)
Please specify the target haematocrit, below which you would be satisfied that venesection was adequate.	__________________________________
Would any of the following features lower your haematocrit threshold / target or push you into considering venesection for this patient?<br><br>History of arterial thrombosis / venous thrombosis / polycythaemia related symptoms / patient request	◯ Yes<br>◯ No

If so, which? (please tick all that apply)	☐ History of arterial thrombosis<br>☐ History of unprovoked venous thrombosis<br>☐ History of provoked venous thrombosis<br>☐ Polycythaemia related symptoms<br>☐ Patient request
Which polycythaemia related symptoms would influence your decision to offer venesection?	☐ Abdominal pain<br>☐ Breathlessness<br>☐ Chest pain<br>☐ Fatigue<br>☐ Headache<br>☐ High blood pressure<br>☐ Light-headedness or dizziness<br>☐ Pruritis<br>☐ Visual disturbance<br>☐ Other (please specify)
Please specify 'other' polycythaemia related symptoms	__________________________________

4. For a patient with unexplained polycythaemia, with extensive work up including bone marrow trephine, red cell mass (true polycythaemia) and negative extended MPN panel and red cell panel i.e. idiopathic erythrocytosis:

Would you offer venesection?	◯ Yes, I would routinely offer venesection.<br>◯ Yes, I would offer venesection for some patients depending on certain clinical features.<br>◯ No I would not offer venesection in any circumstances.
What is your routine threshold haematocrit for starting venesection?	◯ No threshold, I would not routinely offer venesection<br>◯ Hct ≥ 0.6<br>◯ Hct ≥ 0.55<br>◯ Hct ≥ 0.52<br>◯ Hct ≥ 0.48<br>◯ Other (please specify)
Please specify threshold haematocrit at or above which you would offer venesection.	__________________________________
What is your target haematocrit after venesection?	◯ Target Hct < 0.6<br>◯ Target Hct < 0.55<br>◯ Target Hct < 0.52<br>◯ Target Hct < 0.45<br>◯ Other (please specify)
Please specify the target haematocrit, below which you would be satisfied that venesection was adequate.	__________________________________
Would any of the following features lower your haematocrit threshold / target or push you into considering venesection for this patient?<br><br>History of arterial thrombosis / venous thrombosis / polycythaemia related symptoms / patient request	◯ Yes<br>◯ No

If so, which? (please tick all that apply)	☐ History of arterial thrombosis<br>☐ History of unprovoked venous thrombosis<br>☐ History of provoked venous thrombosis<br>☐ Polycythaemia related symptoms<br>☐ Patient request
Which polycythaemia related symptoms would influence your decision to offer venesection?	☐ Abdominal pain<br>☐ Breathlessness<br>☐ Chest pain<br>☐ Fatigue<br>☐ Headache<br>☐ High blood pressure<br>☐ Light-headedness or dizziness<br>☐ Pruritis<br>☐ Visual disturbance<br>☐ Other (please specify)
Please specify 'other' polycythaemia related symptoms	__________________________________

Future Randomised Trial?

Nearly there! This final section is short and designed to gauge whether there is interest in a randomised controlled trial of venesection for patients with secondary or idiopathic polycythaemia.

Would you be willing to include patients with secondary / idiopathic polycythaemia in a study that randomises them to venesection or no venesection?	◯ Yes<br>◯ No

If so, which of the following hypothetical male patients with secondary / idiopathic polycythaemia would you be willing to enter into a study that randomises them to venesection or no venesection?

Haematocrit >0.6 with no other risk factors for thrombosis	◯ Yes<br>◯ No
Haematocrit >0.6 with previous arterial thrombosis	◯ Yes<br>◯ No
Haematocrit >0.6 with previous venous thrombosis	◯ Yes<br>◯ No
Haematocrit 0.55 - 0.6 with no other risk factors for thrombosis	◯ Yes<br>◯ No
Haematocrit 0.55 - 0.6 with previous arterial thrombosis	◯ Yes<br>◯ No
Haematocrit 0.55 - 0.6 with previous venous thrombosis	◯ Yes<br>◯ No
Patients with symptoms associated with polycythaemia	◯ Yes<br>◯ No
Please let us know why you would not be willing to participate.	__________________________________
Approximately how many (male and female) patients are there per year with secondary / idiopathic polycythaemia who newly present to your centre who you would be willing to enter into such a study?	__________________________________<br>(This information will help with study design and planning the number of sites we approach)
Would you like to be added to a future HaemTRIAL mailing list?	◯ Yes<br>◯ No<br>(This will include updates / communications about this and future trials in medical (non-malignant) haematology)
Do you want to be added to the HaemSTAR mailing list (www.HaemSTAR.org)?	◯ Yes<br>◯ No<br>(This is an email with a frequency of approximately once a month.)

Please give us your email address

____
(Provision of your email address is a sign that you consent your email address being stored by HaemSTAR and HaemTRIAL)
