# Supplementary material for: Common Themes and Uncertainties in Management of Secondary Polycythaemia: An International Clinician Survey of Practice
Source: EJHaem. 2025 Oct 27;6(6):e70171. doi: 10.1002/jha2.70171 (PMC12558438; doi:10.1002/jha2.70171)
Supplement: Supplementary file 2 — Supporting File 2: jha270171‐sup‐0002‐SuppMat.docx [file JHA2-6-e70171-s001.docx]

### Supplementary Material

|  | n | % |
| --- | --- | --- |
| Total respondents | 123 |  |
| Consultants | 90 | 73.2% |
| Registrars / Fellows | 20 | 16.3% |
| Specialty Doctors | 9 | 7.3% |
| CNSs | 4 | 3.3% |
|  |  |  |
| Of total respondents |  |  |
| UK | 101 | 82.1% |
| non-UK | 22 | 17.9% |
|  |  |  |
| Number who regularly venesect for secondary polycythaemia | 47 | 38.2% |
| Number who give cytoreduction to patients with secondary polycythaemia | 12 | 9.8% |
|  |  |  |
| Of consultants |  |  |
| UK | 76 | 84.4% |
| non-UK | 14 | 15.6% |
|  |  |  |
| Number who regularly venesect for secondary polycythaemia | 34 | 37.8% |
| Number who give cytoreduction to patients with secondary polycythaemia | 7 | 7.8% |

**Supplementary Table 1: demographics of respondents**

|  | n | % |
| --- | --- | --- |
| Consultants who answered the cases | 78 | 86.7% |
| Consultants who did not answer the cases | 12 | 13.3% |
|  |  |  |
| **Case 1: Polycythaemia secondary to OSA following optimisation with CPAP** |  |  |
| Consultants who answered cases who answered case 1 | 78 | 100.0% |
| Consultants who answered cases who did not answer case 1 | 0 | 0.0% |
| Consultants who would routinely venesect | 5 | 6.4% |
| Consultants who would venesect in certain circumstances | 46 | 59.0% |
| Consultants who would not venesect at all | 27 | 34.6% |
|  |  |  |
| Consultants who do not have a threshold for starting venesection because they would not routinely do it | 4 | 7.8% |
| Consultants who would start vensection at Hct ≥ 0.6 | 18 | 35.3% |
| Consultants who would start vensection at Hct ≥ 0.55 | 25 | 49.0% |
| Consultants who would start vensection at Hct ≥ 0.52 | 0 | 0.0% |
| Consultants who would start vensection at Hct ≥ 0.48 | 1 | 2.0% |
| Consultants who would start vensection at another venesection target | 3 | 5.9% |
|  |  |  |
| Consultants who said they would venesect and offered a target haematocrit | 47 | 92.2% |
| Consultants who said they would venesect but didn't offer a target haematocrit | 4 | 7.8% |
| Target Hct < 0.6 | 5 | 9.8% |
| Target Hct< 0.55 | 26 | 51.0% |
| Target Hct < 0.52 | 13 | 25.5% |
| Target Hct < 0.45 | 1 | 2.0% |
| Target Hct 'Other" | 2 | 3.9% |
|  |  |  |
| Consultants who answered the question about clinical features would alter Hct target / threshold | 51 |  |
| Consultants who said clinical features would alter venesection target / threshold | 47 | 92.2% |
| Consultants who said clinical features wouldn't alter venesection target / threshold | 4 | 7.8% |
|  |  |  |
| Consultants who said clinical features would alter Hct threshold / target who went on to elaborate on which features | 47 | 92.2% |
| Influencing clinical features: History of arterial thrombosis | 41 | 80.4% |
| Influencing clinical features: History of unprovoked venous thrombosis | 43 | 84.3% |
| Influencing clinical features: History of provoked venous thrombosis | 8 | 15.7% |
| Influencing clinical features: Polycythaemia related symptoms | 36 | 70.6% |
| Influencing clinical features: Patient request | 8 | 15.7% |
| Symptoms: Abdominal Pain | 2 | 3.9% |
| Symptoms: Breathlessness | 7 | 13.7% |
| Symptoms: Chest Pain | 7 | 13.7% |
| Symptoms: Fatigue | 10 | 19.6% |
| Symptoms: Headache | 34 | 66.7% |
| Symptoms: High blood pressure | 8 | 15.7% |
| Symptoms: Light-headedness / dizziness | 16 | 31.4% |
| Symptoms: Pruritus | 21 | 41.2% |
| Symptoms: Visual disturbance | 32 | 62.7% |
| Symptoms: Other | 1 | 2.0% |
|  | | |
| **Case 2: Polycythaemia secondary to COPD following optimisation after Respiratory Consultation** |  |  |
| Consultants who answered cases who answered case 2 | 78 | 100.0% |
| Consultants who answered cases who did not answer case 2 | 0 | 0.0% |
| Consultants who answered who would routinely venesect | 1 | 1.3% |
| Consultants who would venesect in certain circumstances | 47 | 60.3% |
| Consultants who would not venesect at all | 30 | 38.5% |
|  |  |  |
| Consultants who do not have a threshold for starting venesection because they wouldn't 'routinely' do it | 5 | 10.4% |
| Consultants who would start vensection at Hct ≥ 0.6 | 17 | 35.4% |
| Consultants who would start vensection at Hct ≥ 0.55 | 24 | 50.0% |
| Consultants who would start vensection at Hct ≥ 0.52 | 0 | 0.0% |
| Consultants who would start vensection at Hct ≥ 0.48 | 0 | 0.0% |
| Consultants who would start vensection at another venesection target | 2 | 4.2% |
|  |  |  |
| Consultants who said they would venesect and offered a target haematocrit | 43 | 89.6% |
| Consultants who said they would venesect but didn't offer a target haematocrit | 5 | 10.4% |
| Target Hct < 0.6 | 5 | 10.4% |
| Target Hct< 0.55 | 23 | 47.9% |
| Target Hct < 0.52 | 13 | 27.1% |
| Target Hct < 0.45 | 1 | 2.1% |
| Target Hct 'Other" | 1 | 2.1% |
|  |  |  |
| Consultants who answered the question about clinical features would alter Hct target / threshold | 48 |  |
| Consultants who said clinical features would alter venesection target / threshold | 40 | 83.3% |
| Consultants who said clinical features wouldn't alter venesection target / threshold | 8 | 16.7% |
|  |  |  |
| Consultants who said clinical features would alter Hct threshold / target who went on to tell us what features | 40 | 83.3% |
| Influencing clinical features: History of arterial thrombosis | 30 | 62.5% |
| Influencing clinical features: History of unprovoked venous thrombosis | 33 | 68.8% |
| Influencing clinical features: History of provoked venous thrombosis | 11 | 22.9% |
| Influencing clinical features: Polycythaemia related symptoms | 33 | 68.8% |
| Influencing clinical features: Patient request | 6 | 12.5% |
| Symptoms: Abdominal Pain | 2 | 4.2% |
| Symptoms: Breathlessness | 4 | 8.3% |
| Symptoms: Chest Pain | 7 | 14.6% |
| Symptoms: Fatigue | 10 | 20.8% |
| Symptoms: Headache | 30 | 62.5% |
| Symptoms: High blood pressure | 6 | 12.5% |
| Symptoms: Light-headedness / dizziness | 14 | 29.2% |
| Symptoms: Pruritus | 17 | 35.4% |
| Symptoms: Visual disturbance | 28 | 58.3% |
| Symptoms: Other | 1 | 2.1% |
|  | | |
| **Case 3: Polycythaemia secondary to testosterone that has been optimised for symptoms** |  |  |
| Consultants who answered cases who answered case 3 | 78 | 100.0% |
| Consultants who answered cases who didn't answer case 3 | 0 | 0.0% |
| Consultants who answered who would routinely venesect | 7 | 9.0% |
| Consultants who would venesect in certain circumstances | 36 | 46.2% |
| Consultants who would not venesect at all | 35 | 44.9% |
|  |  |  |
| Consultants who do not have a threshold for starting venesection because they wouldn't 'routinely' do it | 3 | 7.0% |
| Consultants who would start vensection at Hct ≥ 0.6 | 8 | 18.6% |
| Consultants who would start vensection at Hct ≥ 0.55 | 29 | 67.4% |
| Consultants who would start vensection at Hct ≥ 0.52 | 1 | 2.3% |
| Consultants who would start vensection at Hct ≥ 0.48 | 0 | 0.0% |
| Consultants who would start vensection at another venesection target | 2 | 4.7% |
|  |  |  |
| Consultants who said they would venesect and offered a target haematocrit | 40 | 93.0% |
| Consultants who said they would venesect but didn't offer a target haematocrit | 3 | 7.0% |
| Target Hct < 0.6 | 3 | 7.0% |
| Target Hct< 0.55 | 24 | 55.8% |
| Target Hct < 0.52 | 12 | 27.9% |
| Target Hct < 0.45 | 0 | 0.0% |
| Target Hct 'Other" | 1 | 2.3% |
|  |  |  |
| Consultants who answered the question about clinical features would alter Hct target / threshold | 43 |  |
| Consultants who said clinical features would alter venesection target / threshold | 38 | 88.4% |
| Consultants who said clinical features wouldn't alter venesection target / threshold | 5 | 11.6% |
|  |  |  |
| Consultants who said clinical features would alter Hct threshold / target who went on to tell us what features | 38 | 88.4% |
| Influencing clinical features: History of arterial thrombosis | 33 | 76.7% |
| Influencing clinical features: History of unprovoked venous thrombosis | 35 | 81.4% |
| Influencing clinical features: History of provoked venous thrombosis | 11 | 25.6% |
| Influencing clinical features: Polycythaemia related symptoms | 28 | 65.1% |
| Influencing clinical features: Patient request | 7 | 16.3% |
| Symptoms: Abdominal Pain | 2 | 4.7% |
| Symptoms: Breathlessness | 4 | 9.3% |
| Symptoms: Chest Pain | 9 | 20.9% |
| Symptoms: Fatigue | 5 | 11.6% |
| Symptoms: Headache | 24 | 55.8% |
| Symptoms: High blood pressure | 4 | 9.3% |
| Symptoms: Light-headedness / dizziness | 14 | 32.6% |
| Symptoms: Pruritus | 15 | 34.9% |
| Symptoms: Visual disturbance | 24 | 55.8% |
| Symptoms: Other | 2 | 4.7% |
|  | | |
| **Case 4. truly unexplained polycythaemia (ie idiopathic)** |  |  |
| Consultants who answered cases who answered case 4 | 78 | 100.0% |
| Consultants who answered cases who didn't answer case 4 | 0 | 0.0% |
| Consultants who answered who would routinely venesect | 18 | 23.1% |
| Consultants who would venesect in certain circumstances | 47 | 60.3% |
| Consultants who would not venesect at all | 13 | 16.7% |
|  |  |  |
| Consultants who do not have a threshold for starting venesection because they wouldn't 'routinely' do it | 8 | 12.3% |
| Consultants who would start vensection at Hct ≥ 0.6 | 10 | 15.4% |
| Consultants who would start vensection at Hct ≥ 0.55 | 39 | 60.0% |
| Consultants who would start vensection at Hct ≥ 0.52 | 4 | 6.2% |
| Consultants who would start vensection at Hct ≥ 0.48 | 3 | 4.6% |
| Consultants who would start vensection at another venesection target | 1 | 1.5% |
|  |  |  |
| Consultants who said they would venesect and offered a target haematocrit | 57 | 87.7% |
| Consultants who said they would venesect but didn't offer a target haematocrit | 8 | 12.3% |
| Target Hct < 0.6 | 3 | 4.6% |
| Target Hct< 0.55 | 26 | 40.0% |
| Target Hct < 0.52 | 15 | 23.1% |
| Target Hct < 0.45 | 11 | 16.9% |
| Target Hct 'Other" | 2 | 3.1% |
|  |  |  |
| Consultants who answered the question about clinical features would alter Hct target / threshold | 65 |  |
| Consultants who said clinical features would alter venesection target / threshold | 58 | 89.2% |
| Consultants who said clinical features wouldn't alter venesection target / threshold | 7 | 10.8% |
|  |  |  |
| Consultants who said clinical features would alter Hct threshold / target who went on to tell us what features | 58 | 89.2% |
| Influencing clinical features: History of arterial thrombosis | 55 | 84.6% |
| Influencing clinical features: History of unprovoked venous thrombosis | 51 | 78.5% |
| Influencing clinical features: History of provoked venous thrombosis | 16 | 24.6% |
| Influencing clinical features: Polycythaemia related symptoms | 41 | 63.1% |
| Influencing clinical features: Patient request | 7 | 10.8% |
| Symptoms: Abdominal Pain | 6 | 9.2% |
| Symptoms: Breathlessness | 6 | 9.2% |
| Symptoms: Chest Pain | 14 | 21.5% |
| Symptoms: Fatigue | 9 | 13.8% |
| Symptoms: Headache | 35 | 53.8% |
| Symptoms: High blood pressure | 9 | 13.8% |
| Symptoms: Light-headedness / dizziness | 19 | 29.2% |
| Symptoms: Pruritus | 21 | 32.3% |
| Symptoms: Visual disturbance | 40 | 61.5% |
| Symptoms: Other | 1 | 1.5% |

**Supplementary Table 2: consultant responses to hypothetical secondary / idiopathic polycythaemia cases**

|  | n | % |
| --- | --- | --- |
| All who answered the cases | 103 | 83.7% |
| All who did not answer the cases | 20 | 16.3% |
|  |  |  |
| **Case 1: Polycythaemia secondary to OSA following optimisation with CPAP** |  |  |
| All who answered cases who answered case 1 | 103 | 100.0% |
| All who answered cases who didn't answer case 1 | 0 | 0.0% |
| All who answered who would routinely venesect | 5 | 4.9% |
| All who would venesect in certain circumstances | 66 | 64.1% |
| All who would not venesect at all | 32 | 31.1% |
|  |  |  |
| All who do not have a threshold for starting venesection because they wouldn't 'routinely' do it | 6 | 8.5% |
| All who would start vensection at Hct ≥ 0.6 | 24 | 33.8% |
| All who would start vensection at Hct ≥ 0.55 | 37 | 52.1% |
| All who would start vensection at Hct ≥ 0.52 | 0 | 0.0% |
| All who would start vensection at Hct ≥ 0.48 | 1 | 1.4% |
| All who would start vensection at another venesection target | 3 | 4.2% |
|  |  |  |
| All who said they would venesect and offered a target haematocrit | 65 | 91.5% |
| All who said they would venesect but didn't offer a target haematocrit | 6 | 8.5% |
| Target Hct < 0.6 | 8 | 11.3% |
| Target Hct< 0.55 | 31 | 43.7% |
| Target Hct < 0.52 | 22 | 31.0% |
| Target Hct < 0.45 | 2 | 2.8% |
| Target Hct 'Other" | 2 | 2.8% |
|  |  |  |
| All who answered the question about clinical features would alter Hct target / threshold | 71 |  |
| All who said clinical features would alter venesection target / threshold | 63 | 88.7% |
| All who said clinical features wouldn't alter venesection target / threshold | 8 | 11.3% |
|  |  |  |
| All who said clinical features would alter Hct threshold / target who went on to tell us what features | 63 | 88.7% |
| Influencing clinical features: History of arterial thrombosis | 55 | 77.5% |
| Influencing clinical features: History of unprovoked venous thrombosis | 57 | 80.3% |
| Influencing clinical features: History of provoked venous thrombosis | 13 | 18.3% |
| Influencing clinical features: Polycythaemia related symptoms | 50 | 70.4% |
| Influencing clinical features: Patient request | 14 | 19.7% |
| Symptoms: Abdominal Pain | 4 | 5.6% |
| Symptoms: Breathlessness | 12 | 16.9% |
| Symptoms: Chest Pain | 13 | 18.3% |
| Symptoms: Fatigue | 15 | 21.1% |
| Symptoms: Headache | 46 | 64.8% |
| Symptoms: High blood pressure | 12 | 16.9% |
| Symptoms: Light-headedness / dizziness | 24 | 33.8% |
| Symptoms: Pruritus | 30 | 42.3% |
| Symptoms: Visual disturbance | 42 | 59.2% |
| Symptoms: Other | 1 | 1.4% |
|  | | |
| **Case 2: Polycythaemia secondary to COPD following optimisation after Respiratory Consultation** |  |  |
| All who answered cases who answered case 2 | 103 | 100.0% |
| All who answered cases who didn't answer case 2 | 0 | 0.0% |
| All who answered who would routinely venesect | 1 | 1.0% |
| All who would venesect in certain circumstances | 64 | 62.1% |
| All who would not venesect at all | 38 | 36.9% |
|  |  |  |
| All who do not have a threshold for starting venesection because they wouldn't 'routinely' do it | 6 | 9.2% |
| All who would start vensection at Hct ≥ 0.6 | 21 | 32.3% |
| All who would start vensection at Hct ≥ 0.55 | 34 | 52.3% |
| All who would start vensection at Hct ≥ 0.52 | 2 | 3.1% |
| All who would start vensection at Hct ≥ 0.48 | 0 | 0.0% |
| All who would start vensection at another venesection target | 2 | 3.1% |
|  |  |  |
| All who said they would venesect and offered a target haematocrit | 59 | 90.8% |
| All who said they would venesect but didn't offer a target haematocrit | 6 | 9.2% |
| Target Hct < 0.6 | 7 | 10.8% |
| Target Hct< 0.55 | 29 | 44.6% |
| Target Hct < 0.52 | 21 | 32.3% |
| Target Hct < 0.45 | 1 | 1.5% |
| Target Hct 'Other" | 1 | 1.5% |
|  |  |  |
| All who answered the question about clinical features would alter Hct target / threshold | 65 |  |
| All who said clinical features would alter venesection target / threshold | 54 | 83.1% |
| All who said clinical features wouldn't alter venesection target / threshold | 11 | 16.9% |
|  |  |  |
| All who said clinical features would alter Hct threshold / target who went on to tell us what features | 54 | 83.1% |
| Influencing clinical features: History of arterial thrombosis | 42 | 64.6% |
| Influencing clinical features: History of unprovoked venous thrombosis | 46 | 70.8% |
| Influencing clinical features: History of provoked venous thrombosis | 17 | 26.2% |
| Influencing clinical features: Polycythaemia related symptoms | 43 | 66.2% |
| Influencing clinical features: Patient request | 10 | 15.4% |
| Symptoms: Abdominal Pain | 4 | 6.2% |
| Symptoms: Breathlessness | 8 | 12.3% |
| Symptoms: Chest Pain | 12 | 18.5% |
| Symptoms: Fatigue | 14 | 21.5% |
| Symptoms: Headache | 39 | 60.0% |
| Symptoms: High blood pressure | 8 | 12.3% |
| Symptoms: Light-headedness / dizziness | 20 | 30.8% |
| Symptoms: Pruritus | 23 | 35.4% |
| Symptoms: Visual disturbance | 37 | 56.9% |
| Symptoms: Other | 1 | 1.5% |
|  | | |
| **Case 3: Polycythaemia secondary to testosterone that has been optimised for symptoms** |  |  |
| All who answered cases who answered case 3 | 103 | 100.0% |
| All who answered cases who didn't answer case 3 | 0 | 0.0% |
| All who answered who would routinely venesect | 10 | 9.7% |
| All who would venesect in certain circumstances | 49 | 47.6% |
| All who would not venesect at all | 44 | 42.7% |
|  |  |  |
| All who do not have a threshold for starting venesection because they wouldn't 'routinely' do it | 6 | 10.2% |
| All who would start vensection at Hct ≥ 0.6 | 9 | 15.3% |
| All who would start vensection at Hct ≥ 0.55 | 40 | 67.8% |
| All who would start vensection at Hct ≥ 0.52 | 2 | 3.4% |
| All who would start vensection at Hct ≥ 0.48 | 0 | 0.0% |
| All who would start vensection at another venesection target | 2 | 3.4% |
|  |  |  |
| All who said they would venesect and offered a target haematocrit | 53 | 89.8% |
| All who said they would venesect but didn't offer a target haematocrit | 6 | 10.2% |
| Target Hct < 0.6 | 3 | 5.1% |
| Target Hct< 0.55 | 32 | 54.2% |
| Target Hct < 0.52 | 16 | 27.1% |
| Target Hct < 0.45 | 1 | 1.7% |
| Target Hct 'Other" | 1 | 1.7% |
|  |  |  |
| All who answered the question about clinical features would alter Hct target / threshold | 59 |  |
| All who said clinical features would alter venesection target / threshold | 49 | 83.1% |
| All who said clinical features wouldn't alter venesection target / threshold | 10 | 16.9% |
|  |  |  |
| All who said clinical features would alter Hct threshold / target who went on to tell us what features | 49 | 83.1% |
| Influencing clinical features: History of arterial thrombosis | 41 | 69.5% |
| Influencing clinical features: History of unprovoked venous thrombosis | 44 | 74.6% |
| Influencing clinical features: History of provoked venous thrombosis | 17 | 28.8% |
| Influencing clinical features: Polycythaemia related symptoms | 35 | 59.3% |
| Influencing clinical features: Patient request | 10 | 16.9% |
| Symptoms: Abdominal Pain | 4 | 6.8% |
| Symptoms: Breathlessness | 7 | 11.9% |
| Symptoms: Chest Pain | 12 | 20.3% |
| Symptoms: Fatigue | 7 | 11.9% |
| Symptoms: Headache | 31 | 52.5% |
| Symptoms: High blood pressure | 7 | 11.9% |
| Symptoms: Light-headedness / dizziness | 17 | 28.8% |
| Symptoms: Pruritus | 19 | 32.2% |
| Symptoms: Visual disturbance | 31 | 52.5% |
| Symptoms: Other | 2 | 3.4% |
|  | | |
| **Case 4: Truly unexplained polycythaemia (ie idiopathic)** |  |  |
| All who answered cases who answered case 4 | 103 | 100.0% |
| All who answered cases who didn't answer case 4 | 0 | 0.0% |
| All who answered who would routinely venesect | 19 | 18.4% |
| All who would venesect in certain circumstances | 67 | 65.0% |
| All who would not venesect at all | 17 | 16.5% |
|  |  |  |
| All who do not have a threshold for starting venesection because they wouldn't 'routinely' do it | 9 | 10.5% |
| All who would start vensection at Hct ≥ 0.6 | 15 | 17.4% |
| All who would start vensection at Hct ≥ 0.55 | 52 | 60.5% |
| All who would start vensection at Hct ≥ 0.52 | 6 | 7.0% |
| All who would start vensection at Hct ≥ 0.48 | 3 | 3.5% |
| All who would start vensection at another venesection target | 1 | 1.2% |
|  |  |  |
| All who said they would venesect and offered a target haematocrit | 77 | 89.5% |
| All who said they would venesect but didn't offer a target haematocrit | 9 | 10.5% |
| Target Hct < 0.6 | 5 | 5.8% |
| Target Hct< 0.55 | 32 | 37.2% |
| Target Hct < 0.52 | 25 | 29.1% |
| Target Hct < 0.45 | 13 | 15.1% |
| Target Hct 'Other" | 2 | 2.3% |
|  |  |  |
| All who answered the question about clinical features would alter Hct target / threshold | 86 |  |
| All who said clinical features would alter venesection target / threshold | 75 | 87.2% |
| All who said clinical features wouldn't alter venesection target / threshold | 11 | 12.8% |
|  |  |  |
| All who said clinical features would alter Hct threshold / target who went on to tell us what features | 75 | 87.2% |
| Influencing clinical features: History of arterial thrombosis | 71 | 82.6% |
| Influencing clinical features: History of unprovoked venous thrombosis | 67 | 77.9% |
| Influencing clinical features: History of provoked venous thrombosis | 27 | 31.4% |
| Influencing clinical features: Polycythaemia related symptoms | 54 | 62.8% |
| Influencing clinical features: Patient request | 12 | 14.0% |
| Symptoms: Abdominal Pain | 8 | 9.3% |
| Symptoms: Breathlessness | 10 | 11.6% |
| Symptoms: Chest Pain | 20 | 23.3% |
| Symptoms: Fatigue | 13 | 15.1% |
| Symptoms: Headache | 46 | 53.5% |
| Symptoms: High blood pressure | 14 | 16.3% |
| Symptoms: Light-headedness / dizziness | 27 | 31.4% |
| Symptoms: Pruritus | 29 | 33.7% |
| Symptoms: Visual disturbance | 52 | 60.5% |
| Symptoms: Other | 1 | 1.2% |

**Supplementary Table 3: all respondents’ responses to hypothetical secondary / idiopathic polycythaemia cases**

|  | n | % |
| --- | --- | --- |
| All who answered the cases | 86 | 85.1% |
| All who did not answer the cases | 15 | 14.9% |
|  |  |  |
| **Case 1: Polycythaemia secondary to OSA following optimisation with CPAP** |  |  |
| All who answered cases who answered case 1 | 86 | 100% |
| All who answered cases who didn't answer case 1 | 0 | 0% |
| All who answered who would routinely venesect | 3 | 3.5% |
| All who would venesect in certain circumstances | 56 | 65.1% |
| All who would not venesect at all | 27 | 31.4% |
|  |  |  |
| All who do not have a threshold for starting venesection because they wouldn't 'routinely' do it | 5 | 8.5% |
| All who would start vensection at Hct ≥ 0.6 | 20 | 33.9% |
| All who would start vensection at Hct ≥ 0.55 | 31 | 52.5% |
| All who would start vensection at Hct ≥ 0.52 | 0 | 0% |
| All who would start vensection at Hct ≥ 0.48 | 0 | 0% |
| All who would start vensection at another venesection target | 3 | 5.1% |
|  |  |  |
| All who said they would venesect and offered a target haematocrit | 54 | 91.5% |
| All who said they would venesect but didn't offer a target haematocrit | 5 | 8.5% |
| Target Hct < 0.6 | 6 | 10.2% |
| Target Hct< 0.55 | 27 | 45.8% |
| Target Hct < 0.52 | 18 | 30.5% |
| Target Hct < 0.45 | 1 | 1.7% |
| Target Hct 'Other" | 2 | 3.4% |
|  |  |  |
| All who answered the question about clinical features would alter Hct target / threshold | 59 |  |
| All who said clinical features would alter venesection target / threshold | 54 | 91.5% |
| All who said clinical features wouldn't alter venesection target / threshold | 5 | 8.5% |
|  |  |  |
| All who said clinical features would alter Hct threshold / target who went on to tell us what features | 54 |  |
| Influencing clinical features: History of arterial thrombosis | 46 | 85.2% |
| Influencing clinical features: History of unprovoked venous thrombosis | 49 | 90.7% |
| Influencing clinical features: History of provoked venous thrombosis | 11 | 20.4% |
| Influencing clinical features: Polycythaemia related symptoms | 41 | 75.9% |
| Influencing clinical features: Patient request | 12 | 22.2% |
| Symptoms: Abdominal Pain | 3 | 7.3% |
| Symptoms: Breathlessness | 8 | 19.5% |
| Symptoms: Chest Pain | 10 | 24.4% |
| Symptoms: Fatigue | 12 | 29.3% |
| Symptoms: Headache | 38 | 92.7% |
| Symptoms: High blood pressure | 7 | 17.1% |
| Symptoms: Light-headedness / dizziness | 18 | 43.9% |
| Symptoms: Pruritus | 23 | 56.1% |
| Symptoms: Visual disturbance | 35 | 85.4% |
| Symptoms: Other | 1 | 2.4% |
|  | | |
| **Case 2: Polycythaemia secondary to COPD following optimisation after Respiratory Consultation** |  |  |
| All who answered cases who answered case 2 | 86 | 100% |
| All who answered cases who didn't answer case 2 | 0 | 0% |
| All who answered who would routinely venesect | 0 | 0% |
| All who would venesect in certain circumstances | 55 | 64% |
| All who would not venesect at all | 31 | 36% |
|  |  |  |
| All who do not have a threshold for starting venesection because they wouldn't 'routinely' do it | 2 | 3.6% |
| All who would start vensection at Hct ≥ 0.6 | 19 | 34.5% |
| All who would start vensection at Hct ≥ 0.55 | 30 | 54.5% |
| All who would start vensection at Hct ≥ 0.52 | 2 | 3.6% |
| All who would start vensection at Hct ≥ 0.48 | 0 | 0% |
| All who would start vensection at another venesection target | 2 | 3.6% |
|  |  |  |
| All who said they would venesect and offered a target haematocrit | 53 | 96.4% |
| All who said they would venesect but didn't offer a target haematocrit | 2 | 3.6% |
| Target Hct < 0.6 | 6 | 10.9% |
| Target Hct< 0.55 | 27 | 49.1% |
| Target Hct < 0.52 | 19 | 34.5% |
| Target Hct < 0.45 | 0 | 0% |
| Target Hct 'Other" | 1 | 1.8% |
|  |  |  |
| All who answered the question about clinical features would alter Hct target / threshold | 55 |  |
| All who said clinical features would alter venesection target / threshold | 47 | 85.5% |
| All who said clinical features wouldn't alter venesection target / threshold | 8 | 14.5% |
|  |  |  |
| All who said clinical features would alter Hct threshold / target who went on to tell us what features | 47 |  |
| Influencing clinical features: History of arterial thrombosis | 37 | 78.7% |
| Influencing clinical features: History of unprovoked venous thrombosis | 41 | 87.2% |
| Influencing clinical features: History of provoked venous thrombosis | 14 | 29.8% |
| Influencing clinical features: Polycythaemia related symptoms | 37 | 78.7% |
| Influencing clinical features: Patient request | 9 | 19.1% |
| Symptoms: Abdominal Pain | 3 | 6.4% |
| Symptoms: Breathlessness | 6 | 12.8% |
| Symptoms: Chest Pain | 9 | 19.1% |
| Symptoms: Fatigue | 12 | 25.5% |
| Symptoms: Headache | 33 | 70.2% |
| Symptoms: High blood pressure | 5 | 10.6% |
| Symptoms: Light-headedness / dizziness | 17 | 36.2% |
| Symptoms: Pruritus | 19 | 40.4% |
| Symptoms: Visual disturbance | 32 | 68.1% |
| Symptoms: Other | 1 | 2.1% |
|  | | |
| **Case 3: Polycythaemia secondary to testosterone that has been optimised for symptoms** |  |  |
| All who answered cases who answered case 3 | 86 | 100% |
| All who answered cases who didn't answer case 3 | 0 | 0% |
| All who answered who would routinely venesect | 7 | 8.1% |
| All who would venesect in certain circumstances | 42 | 48.8% |
| All who would not venesect at all | 37 | 43% |
|  |  |  |
| All who do not have a threshold for starting venesection because they wouldn't 'routinely' do it | 4 | 8.2% |
| All who would start vensection at Hct ≥ 0.6 | 8 | 16.3% |
| All who would start vensection at Hct ≥ 0.55 | 35 | 71.4% |
| All who would start vensection at Hct ≥ 0.52 | 0 | 0% |
| All who would start vensection at Hct ≥ 0.48 | 0 | 0% |
| All who would start vensection at another venesection target | 2 | 4.1% |
|  |  |  |
| All who said they would venesect and offered a target haematocrit | 45 | 91.8% |
| All who said they would venesect but didn't offer a target haematocrit | 4 | 8.2% |
| Target Hct < 0.6 | 2 | 4.1% |
| Target Hct< 0.55 | 29 | 59.2% |
| Target Hct < 0.52 | 13 | 26.5% |
| Target Hct < 0.45 | 0 | 0% |
| Target Hct 'Other" | 1 | 2% |
|  |  |  |
| All who answered the question about clinical features would alter Hct target / threshold | 49 |  |
| All who said clinical features would alter venesection target / threshold | 41 | 87.7% |
| All who said clinical features wouldn't alter venesection target / threshold | 8 | 16.3% |
|  |  |  |
| All who said clinical features would alter Hct threshold / target who went on to tell us what features | 41 |  |
| Influencing clinical features: History of arterial thrombosis | 33 | 80.5% |
| Influencing clinical features: History of unprovoked venous thrombosis | 37 | 90.2% |
| Influencing clinical features: History of provoked venous thrombosis | 14 | 34.1% |
| Influencing clinical features: Polycythaemia related symptoms | 28 | 68.3% |
| Influencing clinical features: Patient request | 9 | 22% |
| Symptoms: Abdominal Pain | 3 | 7.3% |
| Symptoms: Breathlessness | 5 | 12.2% |
| Symptoms: Chest Pain | 9 | 22% |
| Symptoms: Fatigue | 6 | 14.6% |
| Symptoms: Headache | 26 | 63.4% |
| Symptoms: High blood pressure | 5 | 12.2% |
| Symptoms: Light-headedness / dizziness | 13 | 31.7% |
| Symptoms: Pruritus | 13 | 31.7% |
| Symptoms: Visual disturbance | 26 | 63.4% |
| Symptoms: Other | 2 | 4.9% |
|  | | |
| **Case 4: Truly unexplained polycythaemia (ie idiopathic)** |  |  |
| All who answered cases who answered case 4 | 86 | 100% |
| All who answered cases who didn't answer case 4 | 0 | 0% |
| All who answered who would routinely venesect | 15 | 17.4% |
| All who would venesect in certain circumstances | 56 | 65.1% |
| All who would not venesect at all | 15 | 17.4% |
|  |  |  |
| All who do not have a threshold for starting venesection because they wouldn't 'routinely' do it | 5 | 7% |
| All who would start vensection at Hct ≥ 0.6 | 13 | 18.3% |
| All who would start vensection at Hct ≥ 0.55 | 46 | 64.8% |
| All who would start vensection at Hct ≥ 0.52 | 3 | 4.2% |
| All who would start vensection at Hct ≥ 0.48 | 3 | 4.2% |
| All who would start vensection at another venesection target | 1 | 1.4% |
|  |  |  |
| All who said they would venesect and offered a target haematocrit | 66 | 93% |
| All who said they would venesect but didn't offer a target haematocrit | 5 | 7% |
| Target Hct < 0.6 | 4 | 5.6% |
| Target Hct< 0.55 | 30 | 42.3% |
| Target Hct < 0.52 | 22 | 31% |
| Target Hct < 0.45 | 8 | 11.3% |
| Target Hct 'Other" | 2 | 2.8% |
|  |  |  |
| All who answered the question about clinical features would alter Hct target / threshold | 71 |  |
| All who said clinical features would alter venesection target / threshold | 63 | 88.7% |
| All who said clinical features wouldn't alter venesection target / threshold | 8 | 11.3% |
|  |  |  |
| All who said clinical features would alter Hct threshold / target who went on to tell us what features | 63 |  |
| Influencing clinical features: History of arterial thrombosis | 59 | 93.7% |
| Influencing clinical features: History of unprovoked venous thrombosis | 56 | 88.9% |
| Influencing clinical features: History of provoked venous thrombosis | 20 | 31.7% |
| Influencing clinical features: Polycythaemia related symptoms | 43 | 68.3% |
| Influencing clinical features: Patient request | 9 | 14.3% |
| Symptoms: Abdominal Pain | 6 | 9.5% |
| Symptoms: Breathlessness | 5 | 7.9% |
| Symptoms: Chest Pain | 14 | 22.2% |
| Symptoms: Fatigue | 9 | 14.3% |
| Symptoms: Headache | 37 | 58.7% |
| Symptoms: High blood pressure | 9 | 14.3% |
| Symptoms: Light-headedness / dizziness | 18 | 28.6% |
| Symptoms: Pruritus | 21 | 33.3% |
| Symptoms: Visual disturbance | 41 | 65.1% |
| Symptoms: Other | 1 | 1.6% |

**Supplementary Table 4: all UK respondents’ responses to hypothetical secondary / idiopathic polycythaemia cases**

|  | n | % |
| --- | --- | --- |
| Total number responding to future clinical trial question | 101 |  |
|  |  |  |
| total number willing to randomise patients | 86 | 85.1% |
|  |  |  |
| total number willing to randomise patients >0.6 no risk factors | 64 | 74.4% |
| total number willing to randomise patients >0.6 if arterial thrombosis | 60 | 69.8% |
| total number willing to randomise patients >0.6 if venous thrombosis | 59 | 68.6% |
|  |  |  |
| total number willing to randomise patients 0.55 - 0.6 no risk factors | 61 | 70.9% |
| total number willing to randomise patients 0.55 - 0.6 if arterial thrombosis | 65 | 75.6% |
| total number willing to randomise patients 0.55 - 0.6 if venous thrombosis | 64 | 74.4% |

**Supplementary Table 5: respondents’ willingness to engage in a future randomised trial**

**
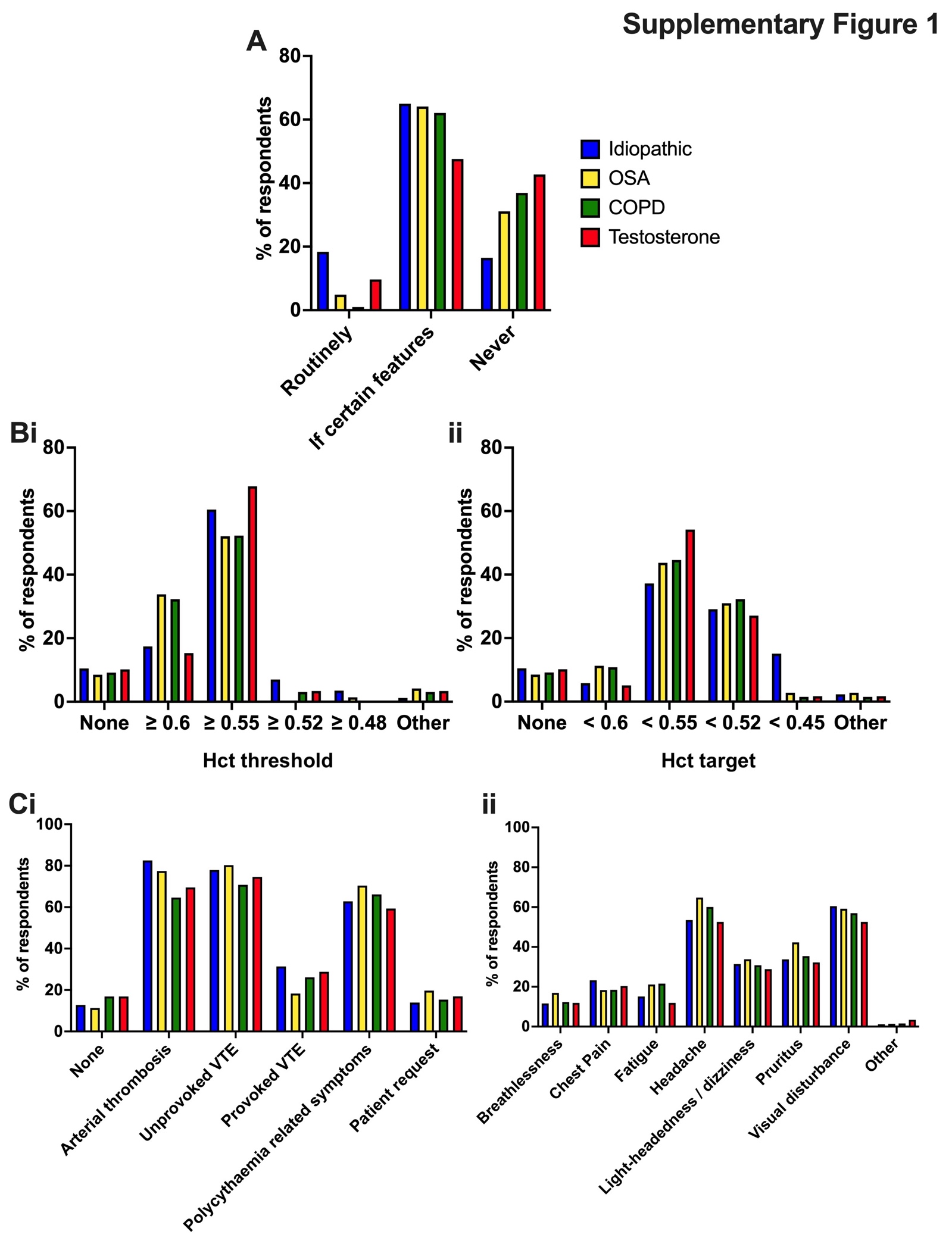

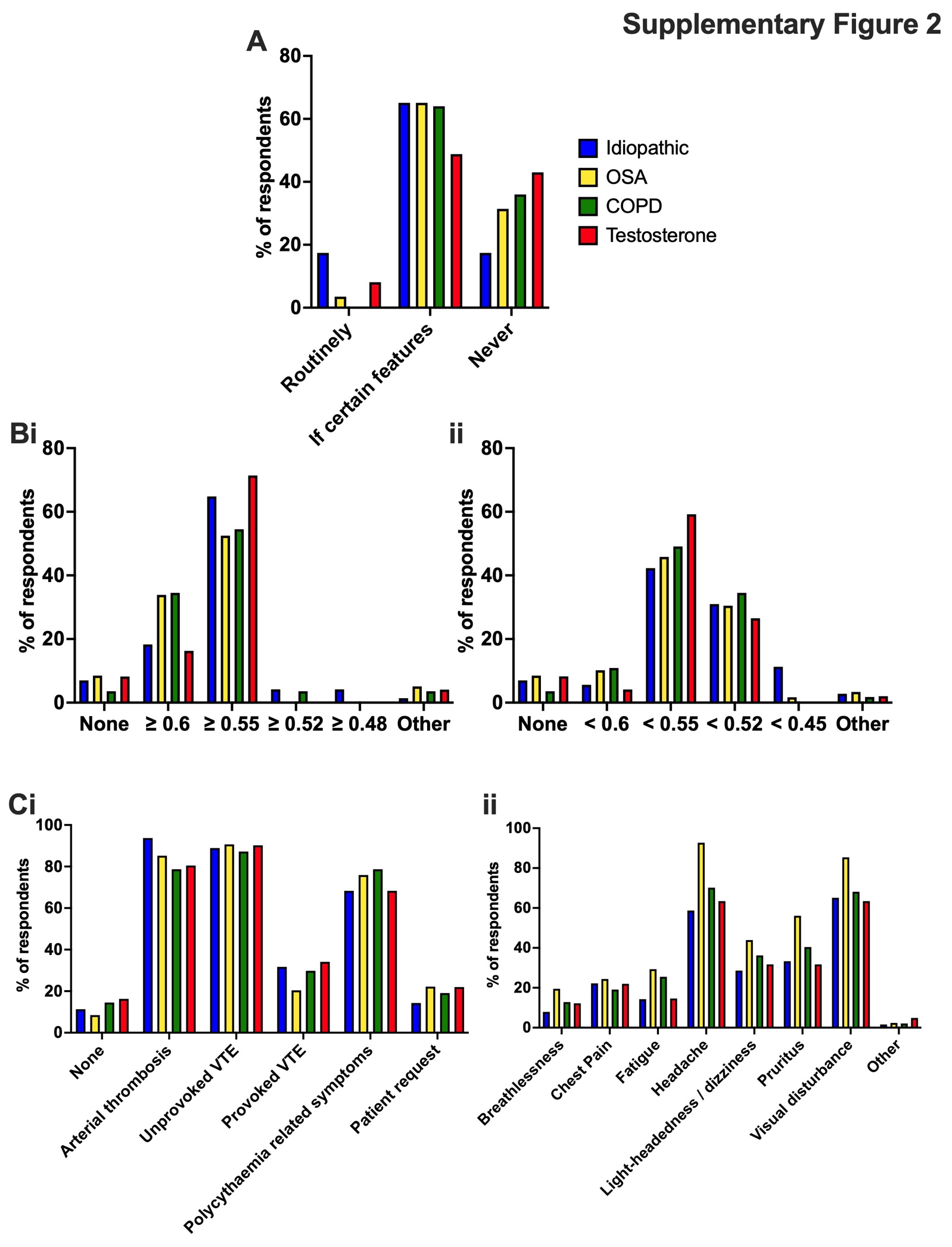
**

**Supplementary Figure 1: Management suggestions on four hypothetical cases of secondary polycythaemia by all respondents.** In all cases the assumptions made were that the cases were secure and the management of the underlying caused had been medically optimised. A) Percentage of total respondents who would routinely venesect, venesect only in certain circumstances or never venesect each of the cases. Bi) Percentage of total respondents who would use different venesection thresholds. ii) Percentage of total respondents who would use different venesection targets. C) Percentage of respondents indicating that clinical features would alter their management who would initiate venesection or alter threshold/target i) in the presence of certain features in the past medical history, ii) in the presence of certain symptoms.

**Supplementary Figure 2: Management suggestions on four hypothetical cases of secondary polycythaemia by all UK respondents.** In all cases the assumptions made were that the cases were secure and the management of the underlying caused had been medically optimised. A) Percentage of total respondents who would routinely venesect, venesect only in certain circumstances or never venesect each of the cases. Bi) Percentage of total respondents who would use different venesection thresholds. ii) Percentage of total respondents who would use different venesection targets. C) Percentage of respondents indicating that clinical features would alter their management who would initiate venesection or alter threshold/target i) in the presence of certain features in the past medical history, ii) in the presence of certain symptoms.
